# Relationship Values and Committed Actions Among Couples Coping with Prostate Cancer: A Qualitative Study

**DOI:** 10.1007/s00520-026-10985-4

**Published:** 2026-07-11

**Authors:** Hongen Ma, Yi Yang, Laura Cariola, David Gillanders

**Affiliations:** 1 Health in Social Science, University of Edinburgh, Edinburgh, UK; 2 Department of Psychiatry, University of Oxford, Oxford, UK

**Keywords:** Prostate cancer, Couples, Partner, Qualitative, Value, Committed actions, Psychological flexibility, Framework method

## Abstract

**Objective:**

Previous research has shown that prostate cancer (PCa) can affect both patients and their partners. However, research into changes in relational dynamics after PCa remains limited. This qualitative study explored the adaptation process through the lens of psychological flexibility (e.g., relationship values and committed actions) in couples affected by PCa.

**Methods:**

Participants were recruited through various strategies, including online advertisements and local community posters. Both in-person and remote individual semi-structured interviews were conducted with eighteen couples (36 interviews). The Framework Method was used to analyse the dyadic interview data qualitatively.

**Results:**

Six main themes and associated sub-themes were generated: (1) psychological responses (initial emotional reactions, recurring thoughts, future perspective, past perspective, present moment awareness), (2) coping styles (responses to thoughts, acceptance, active coping), (3) relationship changes (change in intimacy, relationship re-evaluation, reconnection), (4) relationship barriers (physical challenges, emotional challenges, and social challenges), (5) relationship values (partner identity, partner values, and relationship goals), and (6) relationship commitments (daily activities, planning, partner support).

**Conclusion:**

After PCa diagnosis, couples’ acceptance appears to influence their adaptation processes in terms of psychological adjustment.

**Clinical implications:**

By recognising interpersonal and personal experiences, tailored interventions are needed for both patients and their partners. Promoting psychological flexibility may assist couples in maintaining relationship functioning through psychological and behavioural adaptation during the cancer journey.

**Supplementary Information:**

The online version contains supplementary material available at 10.1007/s00520-026-10985-4.

## Introduction

Prostate cancer (PCa) has become the most common cancer among people assigned male at birth in the UK, with approximately 55,485 new cases diagnosed in 2022 (Prostate Cancer UK, 2025; World Cancer Research Fund 2025). Like other types of cancer, PCa can have a detrimental effect on individuals’ well-being. First, it is important to note that patients may experience various physical side effects following PCa treatments, such as erectile dysfunction, urinary incontinence, and bowel issues (Lane et al. 2022; Sekhoacha et al. 2022). Second, psychological challenges such as anxiety, depression, and low self-esteem are often observed alongside those physical symptoms (Mundle et al. 2021; Scandurra et al. 2022). Additionally, patients’ social connections with their colleagues, friends, and family members may also be affected (Collaço et al. 2021; Salmon et al. 2022). It is evident, therefore, that cancer can have a ripple effect on the well-being of people who have close relationships with patients (van Roij et al. 2019). In particular, the impact of PCa on intimate relationships has attracted growing research attention (Castro et al. 2023).

In previous studies, PCa has been conceptualised as a “we-disease” or “couple-disease” to highlight its relational impact on both patients and their partners (Chambers et al. 2013; Soloway et al. 2005; Wootten et al. 2014a, b). About two-thirds of PCa patients are in an intimate relationship, and most of them have identified their partners as their primary caregivers (Castro et al. 2023; Green et al. 2022). As caregivers, partners may face various burdens, such as providing both emotional and physical support to patients and balancing the demands from work and family (Bijnsdorp et al. 2022; Eriksson et al. 2019). These challenges can lead to physical burnout and emotional distress, which can compromise partners’ well-being (Chong et al. 2023). Additionally, sexual intimacy can be a key aspect of maintaining closeness and satisfaction in a relationship for many couples (Kolodziejczak et al. 2019; Skałacka and Gerymski 2019). However, the physical consequences of PCa and its treatments may lead to substantial disruptions in sexual functioning (Dickstein et al. 2023). If left unaddressed, these disruptions may negatively impact a couple’s ability to maintain emotional and relational intimacy, which can further diminish their relationship satisfaction (Beck et al. 2013; Varner et al. 2019). Therefore, to successfully adapt to life after PCa, it is critical for couples to be mutually aware of the changes introduced by the illness and to assess the newly emerging relational dynamics (Collaço et al. 2021).

Several theoretical models can provide insights into how couples adjust to these changes. According to response shift theory (Schwartz et al. 2007; Sprangers and Schwartz 1999), individuals facing a life-threatening or chronic illness may alter their internal standards, values, and perception of quality of life. These shifts may extend to relationship dynamics in which couples may evaluate their relationships and redefine what matters most to them after PCa. In the cancer survivorship research, the Physical Pleasure-Relational Intimacy Model of Sexual Motivation (PRISM; Beck et al. 2013) offers a nuanced understanding of how couples navigate sexual challenges after cancer treatments. This model suggests that couples who value relational intimacy over physical satisfaction in their sexual experiences are more likely to adapt successfully to changes in sexual function. These findings underline the importance of a couple’s values in shaping their overall adjustment. Notably, the PRISM model identifies acceptance, flexibility, and persistence as key factors in navigating the adjustment process, which aligns with the psychological flexibility model (Hayes et al. 2006, 2012). Psychological flexibility, a core concept within contextual behavioural science, indicates an individual’s ability to focus on the present moment and engage in meaningful actions, even in the face of psychological distress or other unwanted physical symptoms such as pain and fatigue (Carvalho et al. 2022; Gillanders et al. 2024). This model offers a broad lens to examine how couples adapt to PCa both psychologically and behaviourally (Daks and Rogge 2020). There are six components of psychological flexibility: (1) acceptance, (2) defusion, (3) self-as-context, (4) present moment, (5) values, and (6) committed action. The first three components represent the process of psychological adaptation. For example, if patients and their partners can accept the PCa diagnosis without becoming attached to the negative thoughts about it, they are less likely to experience psychological distress (Feros et al. 2013; Serfaty et al. 2019). The last three components depict the behavioural adaptation process. For example, if both patients and partners value being supportive, they are more likely to set goals and take actions to support each other (Lev and McKay 2017). Therefore, an in-depth understanding of psychological flexibility may clarify how couples adapt to life changes and maintain their relationship functioning after PCa.

Psychological flexibility has received growing attention in cancer research as a key factor in promoting adaptability and psychosocial well-being (Chowdhury et al. 2024; Duarte et al. 2023; Hulbert-Williams et al. 2015; McAteer and Gillanders 2019; Sevier‐Guy et al., 2021). However, the dyadic research examining psychological flexibility within couples coping with cancer remains limited. First, most existing cancer studies have focused primarily on the patients, overlooking the experiences and needs of their partners (Gupta et al. 2023; Kleine et al. 2019). Although partners can be affected by PCa, they often receive limited support from the healthcare system, especially during treatment consultations and care planning (Collaço et al. 2018; Li et al. 2022). Second, the interplay between relationship values and committed actions among couples coping with cancer is not well understood. For instance, previous qualitative syntheses (Collaço et al. 2018; Eymech et al. 2022; Green et al. 2022) on PCa patients’ and partners’ experiences highlighted themes such as couples facing cancer together, embarking on new ventures, valuing each day, and gaining new perspectives. While these findings shed light on shared values, there is still limited insight into how couples describe themselves as partners, their relationship goals, and most importantly, the actions guided by their values that they take to maintain their relationships. Furthermore, existing studies that have explored behavioural changes in cancer patients and their partners largely focused on health behaviours, such as quitting smoking, increasing physical activity, or changing dietary habits (Hallward et al. 2020; Midtgaard et al. 2021; Tuinman et al. 2024). Far less is known about specific, value-guided behaviours that couples take to maintain relational connections after PCa. Finally, previous systematic reviews (Chambers et al. 2017a; Ma et al. 2025) have shown that psychosocial interventions aimed at improving relationship functioning among couples coping with PCa have yielded conflicting results. Therefore, there is no clear consensus on the most effective therapeutic approaches. This underscores the need for more targeted research to collect information on patients’ and partners’ experiences and provide insight into developing effective support strategies.

To address this gap, the present qualitative study was designed to develop a deeper understanding of adaptation processes among couples coping with PCa. Based on existing theories and previous study findings, the study sought to answer two key questions. First, what do couples value about their relationships after PCa? Second, what actions do couples take to maintain their relationships? By exploring relationship values and committed actions, this study offers a novel perspective on how couples maintain their relationships throughout the cancer journey.

## Method

### Design

The present study employed a qualitative approach to address the existing research gap by collecting information directly from couples coping with PCa. This approach has been used in previous studies to understand the complex dynamics of relationships and behavioural changes among individuals affected by cancer (Beck et al. 2013; Hallward et al. 2020).

### Participants

To recruit a diverse sample of participants, various recruitment strategies were employed. Couples affected by PCa who responded to the study advertisement online (i.e., Facebook and X) or through the local community posters were invited to participate. Besides the online advertisements and local community posters, patient support organisations were contacted to help post out the study link to their members. Word-of-mouth referrals were also used to encourage participants involved in the study to refer other eligible couples they know who might be interested in participating. To be eligible for the study, couples had to meet the following criteria: (1) at least one partner must be diagnosed with PCa, (2) have been in an intimate relationship for more than 6 months, (3) live and/or receive healthcare in the UK, (4) be aged 18 years or older, and (5) be English-speaking. In this study, partners of PCa patients were defined as individuals in a romantic relationship with the patient, so being a primary caregiver or cohabiting with the patient (living in the same household) was not a requirement for inclusion. Couples were excluded if either partner was unable to participate due to cognitive impairment or other serious health problems. The final sample size was determined when data sufficiency was reached, where no new themes were generated from the individual semi-structured interviews (Hennink et al. 2017).

## Procedure

This study was approved by the ethics committees of the School of Health in Social Science at the University of Edinburgh (23-24CLPS123). As a part of the recruitment process, a Qualtrics online survey link was embedded in the online advertisements, and a QR code was attached to the posters. This allowed interested participants to access the study information sheet, complete an informed consent form, and complete a demographic questionnaire on Qualtrics by clicking the link or scanning the QR code. Therefore, through the Qualtrics survey, participants were aware of the study aims, their ethical rights, and could choose to participate in the interview via phone, video call (i.e., Microsoft Teams), or in person by leaving their contact information for the researcher to schedule the interviews. Consent was obtained separately from each patient and partner, as they signed their own form. Open-ended, semi-structured, one-on-one interviews were conducted, with patients and partners interviewed separately. This allowed each participant to talk about their experiences without their partner influencing their narrative. Following the BPS Code of Ethics and Conduct 2021 (Oates et al. 2021), verbal and recorded consent to allow for audio recording was obtained from all participants before the interview began. All interviews were conducted by the primary investigator (Hongen Ma, pronouns he/him), who had undergone graduate-level training and practice sessions before conducting the semi-structured individual interviews. The interview guide (see Table 1) was developed based on existing theories and models, such as psychological flexibility (Hayes et al. 2012). It included open-ended questions and prompts to encourage participants to talk about their relationship perceptions and committed actions along the cancer journey. All audio recordings were transcribed word for word and checked for accuracy by the team (between Hongen Ma, Laura Cariola, and David Gillanders). Each participant was compensated with a £10 digital gift card upon completion of the interview. Before data analysis, personal information, such as name and identifiable locations, was omitted from the transcripts to maintain confidentiality. Finally, all participants had no prior associations with the research team before participating in the study.

**Table 1:** Interview guide

Focuses	Examples of questions and probes
Diagnosis	What were some of your first reactions when you learned about your/your partner’s diagnosis of prostate cancer?
Relationship	Has your/your partner’s prostate cancer made a difference to how you see your relationship? • Prompt: If so, how would you say your relationship has changed? If you had to describe what your relationship with your partner means to you, what would you say? • Prompt: Any thoughts about what kind of partner you want to be?
Action	On a day-to-day basis, how do you interact with your partner? • Prompt: Do you have particular motivations or inspirations for guiding your actions? Has coping with prostate cancer made a difference to how you choose to act? • Prompt: If so, would you say you are more focused when you spend time with your partner?
Barrier	Is there anything stopping you from staying connected with your partner? • Prompt: Physical? Mental? Emotional?

### Data analysis

The data analysis was conducted by using the Framework Method (Gale et al. 2013; Ritchie and Lewis 2003). Compared to other qualitative methods, such as Grounded Theory or Interpretative Phenomenological Analysis, the Framework Method was deemed more appropriate for this study for three main reasons. First, the decision was based on the development of study questions that were informed by existing theories and models. Second, the Framework Method allowed for a systematic and transparent analysis of collected narratives (Leal et al. 2015). Third, the Framework Method was flexible in terms of allowing comparison across cases for dyadic analysis (Collaço et al. 2021). To better fit the purpose of dyadic focus in the current study, an adapted version of the Framework Method was employed with the additional step of dyadic analysis (Collaço et al. 2021). Thus, the data analysis process consisted of eight stages: (1) transcription, (2) familiarisation, (3) coding, (4) categorising themes, (5) dyadic analysis, (6) developing the analytical framework, (7) applying the framework, and (8) interpreting the data. In the first step, all audio recordings were transcribed word for word into Microsoft Word documents. During the familiarisation stage, transcripts were read and reread to identify any transcription errors and to promote familiarisation with the interview content. Transcripts were then imported into NVivo 15 software for systematic coding. NVivo facilitated the organisation and retrieval of codes by providing a structured environment for managing the data. Once initial coding was complete, all the codes were transferred to the Excel spreadsheet to develop a thematic matrix. This matrix was constructed using both deductive codes generated from interview questions (e.g., relationship values and daily routines) and inductive codes generated directly from participants’ narratives. The Excel spreadsheet was also helpful in displaying the patients’ and partners’ quotes side by side for a better dyadic display for the next step of the dyadic analysis. In this step, dyadic themes, such as relationship barriers and relationship goals, were created based on the patients’ and partners’ narratives. Once preliminary themes were identified from the transcripts of the first four couples, an initial analytical framework was developed. Any questions about the coding decisions and emerging themes were discussed among the research team members to ensure rigour and consistency. The framework was then refined and systematically applied to the remaining transcripts. In the final stage, both individual and dyadic analyses were conducted to explore any patterns within and between patients and partners. Any questions about the interpretation of the findings were discussed among the team members to enhance credibility.

It is important to acknowledge that the entire data analysis process was iterative as we went back and forth through each stage. The first eight transcripts were coded by the first two authors (Hongen Ma and Yi Yang). The codes assigned by each author were compared, and discrepancies were resolved through discussion. Since the two authors’ codes were largely in agreement and did not contradict each other, the remaining 28 transcripts were coded independently by the first author (Hongen Ma). Reflexivity was an important consideration throughout the research process. The interviewer (Hongen Ma) was a young (early 30 s) male researcher who had received training in contextual behavioural science and who had an academic interest in psychological flexibility and dyadic coping. While this background informed the design and theoretical framing of the study, critical reflection was also required during data collection and analysis to minimise potential bias. The gender similarity between the interviewer and male participants may have encouraged open dialogue around sensitive topics such as masculinity and sexual functioning. However, age and professional status differences may have influenced the depth or nature of disclosures, particularly among older participants. To ensure that interpretations remained grounded in the data rather than theory alone, the researcher engaged in reflexive journaling, regular team debriefing, and iterative checking of themes. These strategies were employed to enhance analytical rigour and maintain sensitivity to the diverse perspectives of participants. To write up the manuscript, the Consolidated Criteria for Reporting Qualitative Research (COREQ; Tong et al. 2007) were followed to ensure transparent reporting and methodological rigour.

## Results

### Participants

Initially, 52 potential couples expressed interest in the study by completing consent forms and providing their contact details via Qualtrics. However, only 19 couples responded to interview scheduling attempts. After one couple withdrew due to personal reasons, the final sample consisted of 18 dyads (18 patients and 18 partners). Despite our efforts to recruit a diverse sample through various recruitment strategies, all 18 couples were identified as heterosexual. Patients had a mean age of 68.6 years, and partners’ mean age was 67 years. The average interview duration was 38 min for patients and 36 min for partners. Different PCa stages were reported among patients, including early (*n* = 8), advanced (*n* = 4), and locally advanced (*n* = 6). A range of treatments were received as well, including surgery (*n* = 9), radiation therapy (*n* = 8), hormone therapy (*n* = 8), chemotherapy (*n* = 2), active surveillance (*n* = 4), brachytherapy (*n* = 1), and others (*n* = 2). More detailed demographic information is available in Table 2.

**Table 2:** Demographic information (*N* = 18 Dyads)

Variable	Patient		Partner	
	*M*	*SD*	*M*	*SD*
Age (years)	68.6	8.4	67	9.3
Relationship length (years)*	37.0	17.6	36.7	17.1
Years since diagnosis	4.1	4.3		
	*N*	%	*N*	%
Gender				
Male	18	100.0	0	0.0
Female	0	0.0	18	100.0
Ethnicity				
White	18	100.0	18	100.0
UK areas of residence				
England	13	72.2	13	72.2
Scotland	5	27.8	5	27.8
Sexual orientation				
Heterosexual	18	100.0	18	100.0
Relationship status				
Married, living together	18	100.0	18	100.0
Education				
2-year degree	2	11.1	1	5.6
4-year degree	2	11.1	2	11.1
Doctorate	1	5.6	0	0.0
High school	2	11.1	1	5.6
Less than high school	0	0.0	1	5.6
Professional degree	5	27.8	8	44.3
Some college	6	33.3	5	27.8
Employment				
Disabled	0	0.0	2	11.1
Employed full time	2	11.1	1	5.6
Employed part time	2	11.1	3	16.7
Retired	13	72.2	11	61.0
Not working due to treatment	0	0.0	0	0.0
Unemployed and not looking for work	1	5.6	1	5.6
Psychological support				
No	16	88.9	16	88.9
Yes	2	11.1	2	11.1
Phase of prostate cancer				
Advanced	4	22.2		
Early stage	8	44.5		
Locally advanced	6	33.3		
Treatment type*				
Active surveillance/monitoring	4	22.2		
Brachytherapy	1	5.6		
Chemotherapy	2	11.1		
Hormone therapy	8	44.5		
Other	2	11.1		
Radiation therapy	8	44.5		
Surgery	9	50.0		

*Reported relationship lengths varied among several couple members. Multiple treatments were reported

### Themes

**Fig. 1:**
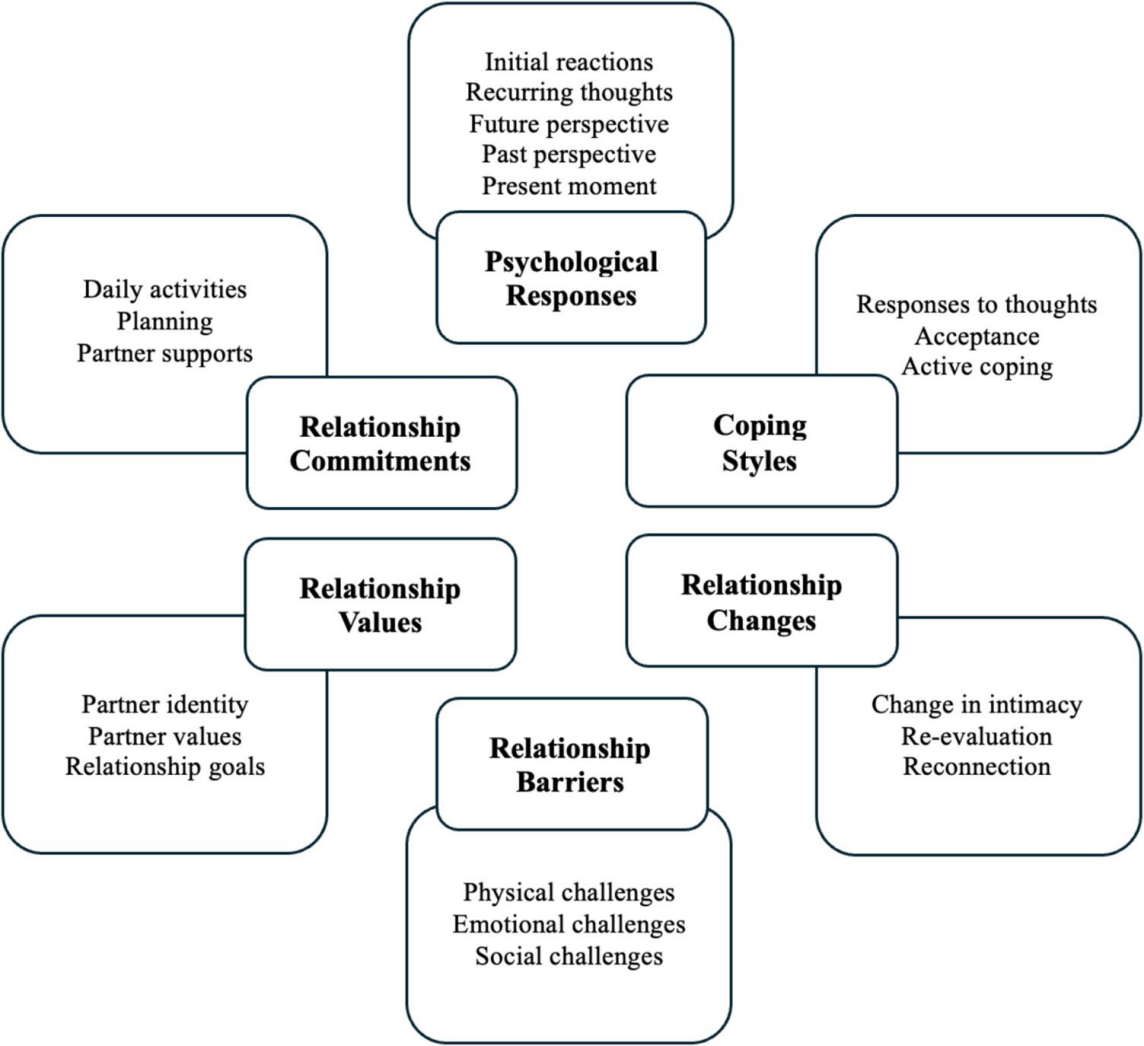
Framework diagram

Based on the analysis, the final analytical framework consisted of 6 main themes and 20 sub-themes (see Fig. 1). Supporting quotes were included to illustrate the themes and ground the findings.

### Theme 1—Psychological responses

The psychological responses observed in this study provided insight into couples’ internal states, including their emotions and thoughts related to PCa. Five sub-themes were identified: initial emotional reactions, recurring thoughts, future perspective, past perspective, and present moment awareness.

#### Initial emotional reactions

Receiving the initial diagnosis of PCa gave rise to a range of emotional responses in both patients and their partners. Patients often reported feeling shocked, surprised, fearful, worried, and nervous. However, some patients also experienced a more moderate response, such as feeling relieved. This was attributed to the confirmation of prior suspicions or seeing the disease as non-threatening.

I don’t remember feeling particularly anxious. I wasn’t waking up at night thinking, “This could be the end.” That thought never crossed my mind. I never saw it as a life-threatening disease. (C2, Patient)

As for partners, the accounts of shock and overwhelming fear were also commonly observed. Some partners associated the diagnosis with death and a gloomy future. One partner in particular expressed the feelings of self-blame.

When it happened, I initially thought it was my fault because there was blood, and we realised something was wrong. As a woman, I’m used to seeing blood, so at first, I assumed it was because of something I had done. But then it became clear that wasn’t the case. I realised that afterwards, but my initial thought was, “Oh dear, my fault.” (C5, Partner)

Yet, moderate emotional responses were also experienced by partners with professional experiences of working in hospitals or with prior experiences related to cancer, such as having lost a loved one to the disease.

#### Recurring thoughts

Both patients and their partners talked about their recurring thoughts about PCa and how it maintained a constant presence in their lives. For patients, these thoughts were primarily triggered by ongoing physical symptoms and medical procedures, such as changes in urine flow, incontinence symptoms, and getting PSA tests. Although some patients noted that these thoughts became less frequent over time, the diagnosis often remained at the back of their minds.

I would say it’s never far away from your mind, yet. It’s a difficult thing to get out of your mind. (C16, Patient)

Partners’ recurring thoughts were mainly triggered by observing the patients’ symptoms and being in the cycle of monitoring, such as seeing patients experiencing hot flushes and sudden fatigue. In particular, partners also cited some external triggers, such as watching news on television and seeing campaign ads on the street. Like patients, partners also expressed a sense that the diagnosis was a permanent fixture and not something that could be entirely forgotten or moved past.

Yeah, I suppose it’s always in the back of my mind. I don’t think about it every day, but I am aware that it could come back. (C14, Partner)

#### Future perspective

Narratives from both patients and their partners revealed how PCa shaped their outlooks on the future. Patients’ future perspectives ranged from anxiety about their remaining time and thoughts about their mortality to having no fears about the future and being confident that things would be fine (‘And it’s also in some ways, you have some sort of recognition of your own mortality’, C11, Patient). Patients also mentioned thinking about personal life goals, such as approaching retirement and spending time with family members. However, partners primarily focused their future concerns on the patients’ health and longevity. They expressed concerns about what would happen to the patients and fears of becoming widowed soon (‘I mean, to start with, I honestly thought I was going to be a widow soon’, C7, Partner). They also hoped that patients’ symptoms could improve in the future.

#### Past perspective

In addition to thoughts about the future, accounts of reflecting on past moments before PCa diagnosis were also noted. Notably, patients reflected on how their physical capabilities used to be, such as their libido and the spontaneity of sex.

Yeah, I would say that I do think about it sometimes. Talking in sexual terms, the spontaneity of sex. That’s not something I can do now. (C12, Patient)

Despite these changes, patients expressed gratitude for past experiences and memories shared with their partners rather than regret. More broadly, one patient thought about his life as a single man before marriage, and another one mentioned that he sometimes thought about his experiences in the military. Partners often spoke about what life was like before the cancer diagnosis, including daily routines and special moments shared together. Both patients and partners acknowledged the irreversible changes brought by PCa and spoke about not dwelling on the past.

Yeah, of course, I wish that things were back the way they were. But I mean, it’s not going to happen, so it’s not a thought I dwell on. (C11, Partner)

#### Present moment awareness

Both patients and their partners talked about how they tried to live in the moment. Patients said that they had decided to live for today because they were aware of how limited their time might be, and because they wanted to enjoy every moment. They were able to ground themselves and focus on the present through daily routines such as work.

Sometimes, if I’m at work and I’m with my colleagues, that keeps me grounded because then I am in the present. (C12, Patient)

Partners also spoke about trying their best to create new shared memories and embrace the present moment.

I know that it’s going to be a point where he won’t be able to do things with us. You know, making memories is going to be more and more difficult as time goes on, and it’s just making those memories. I know we have plenty of memories, but you wanna live in the moment and keep making memories. (C1, Partner)

### Theme 2—Coping styles

Coping styles were explored in this study to understand how couples respond to psychological challenges. Three categories were identified as responses to thoughts, acceptance, and active coping.

#### Responses to thoughts

Both patients and their partners reported diverse reactions to stressful thoughts. They described engaging in open communication about their thoughts and emotions. However, some patients and partners also reported that they tended to keep quiet and keep their thoughts to themselves. A distinction was observed between a proactive response (‘I’m very rational and pragmatic. I process things, and I move forward’, C15, Patient) and a reactive response (‘I probably panic right away. I can be quite pessimistic at first’, C5, Patient). Both patients and partners identified night-time as a difficult time, when stressful thoughts tend to creep in. Hence, they sought distraction by going to bed early or listening to a podcast.

#### Acceptance

Accepting the PCa diagnosis and its consequences was key to couples coming to terms with the disease. Both patients and their partners acknowledged the unchangeable nature of the diagnosis. Patients acknowledged the success of treatments and their personal beliefs, such as destiny and religious faith.

Yes, to an extent, it’s imposed by me not doing enough exercise or not having the right diet or whatever. It has affected my health. Perhaps that’s contributed to the prostate cancer. But I kind of accept that it’s God’s plan for me as a Christian, so. (C10, Patient)

Therefore, they accepted the side effects of treatment and the need for an altered lifestyle. Patients also mentioned the futility of self-pity and preferred a pragmatic approach by accepting the situation, moving forward, and continuing to live their lives. Partners also embraced acceptance, which was associated with prior professional or personal experiences of the disease.

As I said, I was a nurse, and I have seen patients with similar experiences. So, it’s not hard to accept that it happened. (C13, Partner)

Like patients, they noted a shared commitment to making the most of the situation and trying to live life to the fullest.

#### Active coping

Both patients and their partners used various active coping strategies to overcome the psychological challenges associated with PCa. First, open communication with each other was identified as important. Physical activities, such as walking, fishing, and golfing, were also mentioned as helpful ways to cope. Distracting themselves through hobbies, work, and social engagement was another common coping mechanism. While patients and their partners sometimes tended to cope by themselves, partners often hid their own worries to avoid stressing patients. The narratives also highlighted that using humour and practising mindfulness were effective ways of lightening the emotional burden.

Probably tried to make a joke over it and try to discuss it, but at the same time not make it too serious and just try to make light of it. (C15, Partner)

Finally, sharing the pursuit of information through research and joining peer support groups were also observed as helpful strategies to cope with PCa.

One of the things I’ve done since my diagnosis is set up a prostate cancer support group in the county…There were online forums, but I personally found face-to-face conversations more helpful. (C17, Patient)

### Theme 3—Relationship changes

Couples were asked to describe their perceptions of their relationship changes after the PCa diagnosis. In this study, three sub-themes were discovered based on their narratives: change in intimacy, relationship re-evaluation, and reconnection.

#### Change in intimacy

Changes in intimacy, particularly sexual function, were reported by patients and their partners. Patients often mentioned the loss of sexual function, including erectile dysfunction and reduced libido. However, they were still emotionally close to their partners and able to maintain their bond.

I suppose the other part of me is that I mentioned earlier before it changes your physical relationship with your partner. And not everybody is in the same space with that sort of thing. I’m very lucky. I have a close relationship with my wife. (C7, Patient)

Similarly, partners also confirmed that they had stopped having sex. They expressed that they missed their sex life. Yet, they acknowledged the circumstances. Both patients and partners perceived the sexual changes as being associated with their ageing.

When he began having issues with impotence, I was very patient because I know it’s something that happens to men, especially as they get older. (C6, Partner)

#### Relationship re-evaluation

Based on the narratives, the experience of PCa led couples to re-evaluate the importance of their relationships. Patients’ accounts of their experiences after PCa highlighted the importance of their relationships in coping with the disease. They perceived their partners as essential sources of emotional, psychological, and practical support. Without their partners, the patients’ journey would have been difficult.

Oh, there’s no doubt about it. My partner helped me a lot during difficult times. I wouldn’t be here without her. I wouldn’t have had the encouragement, the care, or the support I needed. Psychologically, I would’ve been in an even bigger mess than I was. It would have been almost unbearable. (C5, Patient)

Partners also expressed the significant importance of their relationship. They perceived their relationships as a fundamental source of companionship, friendship, and mutual support during the cancer journey. The cancer experience helped couples to reassess the value of their bond and their strength as a united team.

Neither of us would ever go through a tough time and start wondering, “Are we going to split up?” Some people might think that way when things get difficult, but for us, that doesn’t enter our heads. We just get on with it. You know, we’re a team basically. (C11, Partner)

#### Reconnection

Despite changes in physical intimacy, the experience of PCa promoted a reconnection within couples. Patients reported developing a deeper emotional connection with their partners through increased appreciation and mutual support. Similarly, partners reported a deep appreciation for their relationship. They noted that this experience had improved their communication.

I think, again, it goes back to communication. We’ve always been great communicators with each other, but the depth of our communication and the appreciation for the love we have. It’s grown. I think we count our blessings a lot more now. We genuinely consider ourselves lucky. So yes, I’d say that the level of communication since his diagnosis has definitely deepened. (C7, Partner)

Both patients and their partners said that sharing the experience of PCa had brought them closer and made them stronger.

I think the big change is that our relationship has grown closer. My wife has a better understanding of me, and I’ve developed a better understanding of, well, maybe the situation, if I can put it that way. (C6, Patient)

### Theme 4—Relationship barriers

This study explored relationship barriers to understand the challenges couples experience in maintaining their relationships. These barriers were categorised as physical, emotional or social.

#### Physical challenges

The various physical challenges associated with PCa impacted the daily lives of both patients and their partners. For patients, these included impotence, urinary problems, fatigue, hot flushes, and loss of physical strength. These challenges directly affected their social activities, sleep, and quality of life.

In terms of the physical effects of my treatment, there are some symptoms that cause anxiety for both myself and my partner. For example, I’m now impotent, and I also have diabetes, which complicates things further in the long term. (C14, Patient)

Overall, partners were aware of the physical challenges patients faced and made an effort to support them. They also noted that sleep disturbances were a shared burden, which led to problems with making daily plans.

Yes, neither of us has been sleeping well. That’s been a major issue since the diagnosis. He’s often up in the middle of the night, and I wake up early, around 4 or 5 in the morning. Now, I’m usually up by 5 or 6 at the latest. Ever since we found out, our sleep has been unsettled for both of us. (C18, Partner.)

#### Emotional challenges

In addition to the physical challenges, couples coping with PCa also experienced emotional challenges. Patients struggled with a loss of masculinity and self-esteem due to changes in their sexual abilities.

Yes, I get a bit depressed. I get a bit down. Thinking about that I’m not really a man anymore…I think it’s about self-esteem, possibly masculinity. Yeah, I read this morning in the Telegraph that I seem to be having signs of low testosterone. Low testosterone gives you fatigue and a bit of depression, anxiety, that sort of thing. (C10, Patient)

Patients also experienced anxiety about their own mortality and fear of cancer recurrence. Feelings of depression and mood swings were also reported. Partners, in turn, experienced an emotional burden as they tried to manage their own fears of loss. They noted that they tried to protect the patients’ dignity and self-esteem. Therefore, partners tended to internalise their worries in order to protect the patients from further distress.

Oh, every day. Every day. And sometimes, you know, [partner’s name] doesn’t know this. I don’t share it with him. I don’t want him to know my fear that one day he’s not gonna be here. (C4, Partner)

#### Social challenges

Several external social challenges were also reported, which put pressure on couples’ relationships. Family dynamics were identified as a source of stress. Patients noted that issues with children caused additional worry.

Yeah, we’ve got some issues. I’ve mentioned my oldest daughter. She’s dealing with some problems right now. She and her mother aren’t getting along as well as they should, which is a worry. (C1, Patient)

Partners took on the role of buffering these external stressors and managing difficult family interactions. Financial burdens were also mentioned. For instance, partners sometimes had to take on current financial responsibilities. Furthermore, differences in individual coping mechanisms sometimes led to moments of isolation within couples.

One of my daughters pointed it out to me that we’re, you know, we just seem to be in our own little bubbles and not really talking about it. And I said, well, because we’re probably trying to still process it. (C1, Partner)

### Theme 5—Relationship values

One main goal of this study was to explore relationship values among couples coping with PCa. Based on the narratives, three sub-themes were generated as partner identity, partner values, and relationship goals.

#### Partner identity

The experience of PCa influenced how patients and their partners perceived themselves and each other. Patients described themselves as trying to be supportive and loving. However, they also acknowledged that they were not perfect, such as being impatient, irritable, or distant.

I can’t say yes because I don’t think I’m always fully engaged. The truth is, I’m not the most patient person. It’s not just with my partner. We both don’t suffer fools gladly. If she thinks I’m being difficult, she’ll tell me. I’m not as bold when it comes to doing the same. (C1, Patient)

Patients also recognised the strength of their partners and acknowledged their partners’ important role in helping them to cope with PCa. As for partners, they described themselves as caring, supportive and pragmatic. When partners took on leadership or organisational roles, they sometimes acknowledged that they came across as bossy.

As a partner, I hope I’m supportive. You know, he says that he’d be lost without me. I think I can probably be a bit bossy. But he takes strength from me, and I’ve been with him for every appointment that I think is important. (C7, Partner)

#### Partner values

Accounts from couples about their relationships revealed the core elements they valued most as they navigated the challenges of PCa. Patients said that friendship, open communication, and mutual support formed the foundation of their relationships. They also valued being able to do things together while facing the challenges of ageing with cancer.

The fact that we’re both relatively fit and healthy. And we can still do most of the things we enjoy doing. Not all of them, obviously, but most of the things we enjoy doing, we can still do. (C4, Patient)

Similarly, partners noted that companionship, mutual understanding, loyalty, and open communication were the most important elements of their relationships.

I would say communication and closeness. Being able to talk at all levels with him, and I actually think that I’m very lucky to have that because so many people don’t have that with their partners. (C7, Partner)

They also valued their shared daily life and the support they provided each other.

#### Relationship goals

Couples were asked if they had set themselves any specific goals for their relationship during their cancer journey. Given their age and health status, patients reported that they tended to set short-term goals, such as living in the present, spending more time with family, enjoying shared activities, maintaining their health and staying active.

So, there are many things that stay on our minds. Our main goals are to stay healthy and keep active. Even though I admit I’m a bit lazy with exercise, we do make sure to take steps together. You know, we’re always planning something for the family or organising the next holiday. (C14, Patient)

Their partners’ relationship goals were similar, including having more shared experiences such as travelling, spending more time with family, and improving communication. One partner in particular talked about the goal of restoring their sex life.

It would be quite nice if he could get an erection again and we could have proper sex. That would be good. And that’s what the doctors are now trying to help him with. (C15, Partner)

### Theme 6—Relationship commitments

This study also aimed to explore the committed actions couples take to maintain their relationship after PCa. Three categories were identified as daily activities, planning, and partner support.

#### Daily activities

 Couples reported how they managed their daily lives and shared routines following the PCa diagnosis. Both patients and their partners described spending a lot of time together doing various things, such as cooking, grocery shopping, and having meals together throughout the day. They also mentioned leisure activities, such as walking, watching television, and tending to gardens.

Well, obviously, we like gardening, and we’ve got a nice garden which takes a lot of our time, which we enjoy doing. We have a little dog, and we take the dog out every day. (C4, Patient)

Although patients experienced some physical limitations with certain activities, partners were able to provide some support to help patients.

So, we try and share things, but he’s noticing that he can’t stand up for too long and also with cooking, with the temperature that he can’t cope with the heat. So, I have to sometimes help him out. (C1, Partner)

Taking care of grandchildren and receiving family visits were also part of their regular routines.

#### Planning

Based on the narratives, it was evident that couples continued to make plans for the future following the PCa diagnosis. Both patients and their partners planned for holidays and travel. As patients acknowledged their limited time, they shifted their focus from long-term ambitions to enjoying the present. Partners expressed a preference for doing things sooner rather than later due to treatment schedules and physical limitations.

I think maybe before the prostate cancer, we were sort of avoiding just putting up with it and avoiding it, but now we’re really actively trying to, you know, find somewhere else to live and get sort of excited about that. (C12, Partner)

In addition to leisure activities, planning also involved making practical arrangements for the future, such as discussing housing and even preparing wills.

We’re putting things together, wills and power of attorney and all the things that go with dying, which we always had in the back of our mind. But now it’s come to the front. So now we’re just making certain that what can be in place is in place. (C1, Patient)

#### Partner support

A reciprocal commitment to supporting each other was observed among couples coping with PCa. Patients noted that they supported their partners during difficult times, such as by providing travel assistance after a family loss. They continued to share household responsibilities despite their physical limitations. To prepare their partners for potential incidents, patients actively participated in planning, including important discussions about finances and wills. Patients expressed their support through actions such as hugging, holding hands, and telling their partners how much they cared.

As I mentioned, I’m demonstrative, so I’ll go over and give her a hug, sit beside her, or hold her hand when we’re walking, or just tell her I care. (C14, Patient)

Partners similarly described supportive actions they took, such as researching treatment options, managing medications, taking on increased household responsibilities, and adapting daily routines to ease patients’ burdens.

I’m trying to take on more responsibility because we have horses and dogs. And I’m trying to take on quite a big garden. And I’m trying to make sure that [partner’s name] does less of the work and I do more of the work. (C1, Partner)

## Discussion

To our knowledge, this is the first study to explore both psychological and behavioural adaptations with a focus on relationship values and committed actions among couples coping with PCa. Using a qualitative design, detailed accounts of both patients’ and their partners’ perspectives on how they perceived their relationships and what actions they took to maintain their connections were collected. Following the framework analysis method, six main themes were generated: (1) psychological responses (initial emotional reactions, recurring thoughts, future perspective, past perspective, present moment awareness), (2) coping styles (responses to thoughts, acceptance, active coping), (3) relationship changes (change in intimacy, relationship re-evaluation, reconnection), (4) relationship barriers (physical challenges, emotional challenges, and social challenges), (5) relationship values (partner identity, partner values, and relationship goals), and (6) relationship commitments (daily activities, planning, partner support). The important findings of this study and their implications for cancer care are discussed below.

Several findings from this study align with previous qualitative studies. First, similar to research on responses following a cancer diagnosis (Kirby et al. 2020; Postavaru et al. 2023), our findings suggest that patients and their partners may have different reactions to a cancer diagnosis. These reactions can range from shock and surprise to more moderate responses, such as feeling unruffled or relieved. These differences may be attributed to social or life contexts, such as professional backgrounds and personal beliefs. Additionally, the recurring thoughts triggered by physical symptoms and medical checkups correspond to findings on intrusive thoughts in cancer research (Almeida et al. 2019; Mutsaers et al. 2016; Zhang et al. 2022). Second, the reported physical challenges among patients, such as impotence, urinary issues, and fatigue, are well-studied side effects of PCa treatments (Fox et al. 2019; Sutton et al. 2021). In particular, the findings that partners observed patients struggling with physical challenges and helped them manage these challenges support existing research on caregiver strain (Green et al. 2022; Pinks et al. 2018). Third, the emotional difficulties addressed by patients, such as the loss of masculinity and low self-esteem associated with sexual dysfunction, are commonly studied effects of PCa on male identity (Bowie et al. 2022; Chambers et al. 2017b). Although the findings indicate partners are aware of the impact of PCa on patients’ sense of masculinity, they may not know how to address this issue effectively (Green et al. 2022). Furthermore, similar to previous studies (Walker and Robinson 2011; Wootten et al. 2014a, b), this study observed that couples took the approach of acceptance when facing life changes brought by PCa, particularly regarding the changed sexual activity. Especially, the acceptance of changes in sexual activity may be linked to the ageing process, menopause, or other medical conditions (Chambers et al. 2018; Williams et al. 2014; Wittmann et al. 2015). In addition, consistent with existing research, both patients and partners used specific coping strategies, including open communication, a positive mindset toward PCa, and distraction through busy work lives (Collaço et al. 2021; Spendelow et al. 2018; Wootten et al. 2014a, b). An interesting finding of this study is that humour was also employed as a strategy by patients and their partners to adjust to sexual changes and to maintain positivity by carrying on as normal (Branney et al. 2014; Collaço et al. 2021; Oliffe et al. 2009). The social challenges identified in this study, such as the impact of PCa on work and finances, are also recognised barriers in existing literature (Grunfeld et al. 2013; Timmons et al. 2013). Additionally, the findings indicate that patients and partners may protect one another from distress to prevent further worry, with partners sometimes hesitating to express physical needs and patients fearing that they might not meet expectations (Dieperink et al. 2016; Walker and Robinson 2011; Williams et al. 2014).

In addition to building upon existing knowledge, this study addresses research gaps by exploring how couples define their roles, set relationship goals, and take committed actions to maintain their bond throughout the cancer journey. Consistent with previous research (Albaugh et al. 2017; Collaço et al. 2018; Ervik et al. 2013), our findings reveal that the experience of PCa may bring patients and their partners closer, particularly on an emotional level. Patients’ and partners’ accounts of the importance of their relationships, such as their concern that they may not cope well without each other, highlight the interdependence within the adaptation process. Most importantly, how patients and their partners describe themselves and each other sheds light on the qualities they want in a partner. Words commonly reported, such as supportive, caring, and loyal, suggest that both patients and partners value positive partner qualities. Shared positive self-concepts and identities may promote self-esteem and build psychological resilience, particularly among partners who actively take on caregiving roles. Our findings also show that, when facing life-altering challenges, couples tend to value fundamental aspects such as friendship, open communication, and unwavering mutual support. Although sex can be an important way for couples to maintain intimacy, other aspects of a relationship may be essential for couples to maintain their bond, especially in terms of adjusting to life after PCa (Beck et al. 2013; Collaço et al. 2018). Thus, understanding what couples truly value can provide insight into what kind of support they need the most. In addition, the way patients and partners perceive themselves and their relationship values can be reflected in their behaviours. First, the fact that patients and their partners continue to set goals, particularly short-term ones, suggests that goal setting provides an important sense of purpose and hope. It can act as a buffer against challenges and help couples to maintain their motivation to be active. Second, although daily activities can be spontaneous, couples may encounter obstacles when trying to be active after PCa. Narratives from patients and their partners regarding their joint participation in daily activities, ranging from routine chores to leisure pursuits, demonstrate that couples can remain committed to taking action to cultivate normality and stability, which may help them to avoid isolation and maintain relationship satisfaction. Third, the findings show that patients and their partners can engage in active future planning, including travel and practical arrangements. This may foster a sense of control and readiness in patients and partners, which may reduce their anxiety and uncertainty. Finally, accounts of supportive behaviours, from having more conversations to attending medical appointments together, indicate that patients’ and partners’ actions align with their values of being supportive and caring.

A closer look at our findings suggests that couples coping with PCa may experience a range of challenges both personally and interpersonally. These experiences may be shaped by the different roles that patients and partners assume as they navigate PCa together. At the personal level, patients who were directly affected by PCa described emotional struggles related to physical changes such as impotence and shifts in their sense of masculine identity. Partners, on the other hand, appeared to carry a different emotional burden, which was characterised by concerns about loss and the practical and emotional demands of caregiving. Particularly, it was discovered that patients and partners tended to internalise their distress. This tendency to avoid open emotional expression may be influenced by a variety of factors, including fear of upsetting the other person, feelings of helplessness, and broader social expectations around expressing vulnerability (Collaço et al. 2018). On an interpersonal level, couples used “we” expressions when describing their experiences, which reflected a sense of shared coping. This finding is consistent with previous characterisations of PCa as a “we-disease” (Chambers et al. 2013; Soloway et al. 2005; Wootten et al. 2014a, b). Based on couples’ reports on how they worked together in managing treatment, daily routines, and planning for the future, this shared perspective seemed to foster emotional closeness and collaboration. Overall, these findings highlight the importance of recognising both the individual and relational aspects of coping with PCa. A better understanding of these dynamics may be helpful in guiding future psychosocial support efforts aimed at addressing the needs of both members of the couple.

Overall, the study’s findings connect with the aforementioned theories and models. First, the changes in patients’ and partners’ perspectives on their relationships correspond to the response shift theory (Schwartz et al. 2007; Sprangers and Schwartz 1999). Patients’ and partners’ narratives of how they navigated challenges after PCa, particularly the challenges of sex, reveal the need to reassess the meaning of being in a relationship and adjust how they engage with each other to maintain their bond. Second, while no patient or partner explicitly mentioned valuing sex as the most important aspect in their relationship, they expressed missing the spontaneity of sex and acknowledged that there was not much they could do about it. Taking an acceptance approach that emphasises relational intimacy between patients and partners is consistent with the PRISM model’s suggestion that placing greater value on relational intimacy than physical intimacy may promote more successful adaptation to sexual changes after PCa (Beck et al. 2013). Moreover, this study’s analytic framework is in line with the psychological flexibility model (Hayes et al. 2006, 2012). In the context of acceptance, both patients and their partners recognised the unchangeable nature of the PCa and its consequences. Their commitments to relationship goals and engagements in daily activities while facing physical and emotional challenges, such as travelling, spending time with family, and pursuing shared hobbies, exemplify values-guided behaviours. Not dwelling on the past and trying to make every moment count also reflects cognitive defusion and present-moment awareness. Therefore, our findings indicate that psychological flexibility may be a helpful factor in understanding how couples adapt to life after PCa.

### Limitations and future directions

There are some limitations of this study that need to be addressed. First, all the couples who participated in this study were white, heterosexual, and UK residents. Therefore, our study findings may not apply to couples from a more diverse range of ethnic backgrounds or in other types of relationships. For example, same-sex couples may have different perspectives on the importance of physical intimacy from heterosexual couples. Hence, future studies could include a more diverse sample of participants coping with PCa, such as same-sex couples or couples from non-Western cultures. Second, our findings may not fully represent the experiences of couples who were unwilling to participate in this study. In general, individuals who are more open to sharing their experiences or who have better communication in their relationships may have been more inclined to participate. Consequently, couples who experience communication difficulties or relationship strain may be underrepresented. Since our study participants were mainly recruited through community posters, social media, and referrals, they may experience a lower level of relationship dissatisfaction than nonparticipants. This may have limited the diversity of relational experiences captured in the data. Future studies could use a wider range of recruitment methods, such as clinical settings or targeted outreach, to include a more diverse sample of couples, especially those who are less likely to volunteer for research. Third, patients’ and partners’ narratives need to be interpreted contextually. Although both conjoint and individual interview formats have been used in previous qualitative research (Collaço et al. 2021; Hallward et al. 2020; Manne et al. 2012), this study adapted the individual format by having patients and their partners interviewed separately to ensure each partner’s answers would not be biased. It is possible that patients and partners may have answered differently if they were interviewed together. As aforementioned, the interviewer was a young (early 30 s) male researcher, so it is also possible that participants may have answered differently if they were interviewed by an interviewer who was closer to their age or was of a different gender to them. Given our attempt to encourage participants to speak freely and the level of detail provided, it is very unlikely that participants were understating. Another limitation of the current study is that we did not systematically examine how the experiences of couples varied according to key clinical factors such as PCa stage, treatment modality or time since treatment. These factors may contribute to variations in couples’ accounts of their psychosocial and relational challenges. While the current study aimed to gain a broad understanding of how couples adapt to PCa, future research should adopt a more specific approach to explore how these clinical factors influence couples’ experiences. This would help clinicians to identify the settings and patient populations in which the reported psychosocial and relational challenges are most likely to emerge and to determine where targeted support interventions may be particularly beneficial. Furthermore, our findings may not apply to couples coping with other types of cancer. Since our findings focused on the experiences of heterosexual couples coping with PCa, in this case, patients were male due to the nature of the cancer, and their partners were female. Different dynamics can arise not only in same-sex relationships but also when patients are female and their partners are male (e.g., breast and ovarian cancers). Therefore, future research could explore how relationship values and committed actions differ among couples coping with different types of cancer.

### Clinical implications

The findings of this study have several important clinical implications for supporting couples affected by PCa. While both patients and partners reported receiving adequate information about PCa tests and treatment options, they also expressed unmet needs regarding access to psychosocial support. This highlights the need to improve access to psychosocial interventions to make them more easily available to couples affected by PCa. On the one hand, the discovery of interpersonal experiences of PCa suggests that healthcare providers should recognise the role of partners as an equally impacted and essential part of the care unit. On the other hand, the discovery of personal experiences of PCa indicates that it is also crucial to develop tailored interventions to address the unique needs of both patients and partners. Furthermore, interventions that promote psychological flexibility may be effective in supporting couples to ensure successful adaptation after PCa. Acceptance and not getting too entangled in tricky thoughts and feelings can be the key for couples to psychologically adapt to the unchangeable nature of PCa diagnosis and its consequences. Addressing relationship values and promoting committed actions may help couples to build a sense of motivation, overcome obstacles, and maintain their bond. Therefore, interventions, such as Acceptance and Commitment Therapy (Hayes et al. 2006, 2012), can be adopted by healthcare providers to support couples affected by PCa. Although our data collection took place after the outbreak of the COVID-19 pandemic, most participants opted for remote interviews conducted via telephone or Teams. Some participants noted that they had to learn how to use digital communication tools such as FaceTime and Zoom to maintain social connections during quarantine. This suggests that the remote delivery of psychosocial support (e.g., online ACT) might be a feasible approach for couples coping with PCa.

## Conclusion

Couples may experience changes in their relationship dynamics after PCa. They may choose to accept these changes and focus on living in the moment. Besides physical intimacy, couples may value other aspects of their relationships, such as companionship, open communication, and mutual support. These relationship values can manifest as committed actions to maintain connection, such as sharing daily activities, making plans, and supporting each other. Although both patients and their partners can be affected by PCa, it is important to recognise their unique experiences and needs. Interventions promoting psychological flexibility may support patients’ and partners’ psychological and behavioural adaptations to life after PCa and maintain their relationship functioning.

## Supplementary Information

Below is the link to the electronic supplementary material.

ESM 1 (DOCX 24.2 KB)

## Data Availability

The authors confirm that the data are provided within the manuscript or supplementary information files.
