# Supplementary material for: Relationship Values and Committed Actions Among Couples Coping with Prostate Cancer: A Qualitative Study
Source: Support Care Cancer. 2026 Jul 11;34(8):755. doi: 10.1007/s00520-026-10985-4 (PMC13356049; doi:10.1007/s00520-026-10985-4)
Supplement: Supplementary file 1 — (DOCX 24.2 KB) [file 520_2026_10985_MOESM1_ESM.docx]

Additional supporting quotes

| Subthemes | Quotes |
| --- | --- |
| Initial emotional reactions - emotional reactions first time after hearing about the diagnosis | “There was the usual mix of surprise, disappointment, and a bit of anxiety. I was also quite frustrated with my general practitioner. When I first raised my concerns, I felt belittled and dismissed…I never felt completely overwhelmed by it all, but I was definitely more anxious.” (C14, Patient)  “Trying to think back to that. Yeah, I think upset, clearly upset. A little bit of apprehension. Fear about what our future might bring. Yes, I think life was going along very nicely, and then, all of a sudden, you get hit with curveballs, things you never really expect.” (C14, Partner) |
| Recurring thoughts - frequent thoughts about the diagnosis of PCa | “Oh, yes, yes. I mean it very much took over a lot of my conscious time. I was very, very aware of it every day.” (C8, Patient)  “So you go through phases where it’s not on your mind, and then suddenly, around the time of the PSA test, it comes rushing back. I don’t think the feeling that the cancer could return ever fully goes away.” (C8, Partner) |
| Future perspective - frequent thoughts about what is going to happen in the future | “I worry, am I gonna be alive if I have time, you know? Am I going to be alive in a year’s time? You know, you never know what’s going to happen.” (C10, Patient)  “Am I worried about the future? I don’t think I’m worried about the future. I’m anxious that we make the right plans for the future. You know, you sell the big house in time. You’re downsizing in time. You use your money wisely in time.” (C10, Partner) |
| Past perspective - frequent thoughts about things happened before the diagnosis | “There are moments where I think, what if I hadn’t done this or that? Maybe I wouldn’t have gotten cancer. But I don’t dwell on it much anymore. I’m just grateful to be here.” (C5, Patient)  “No, I remember the past. Happy and then sad that those special times are not available to us, but I remember them with great happiness and gratitude. As I said, we are trying to make the most of our relationship at this moment. Whatever happened, happened.” (C5, Partner) |
| Present moment awareness - the ability to focus on the present moment | “And you know, we try to enjoy every moment. Well, we try to go out as much as we can, add meals out and play and book vacations and go on vacations and just say in general because obviously, we know that my cancer could rear its head at any time. So, I don’t know how long I have. So, we try to enjoy it as much as we can.” (C4, Patient)  “No, no, not at all. We’ve got a good relationship. You know we still hold hands when we go out. No, I don’t have a problem being with my husband. No, not at all. We want to enjoy every moment we have together.” (C4, Partner) |
| Responses to thoughts - how couples respond to stressful thoughts | “Sometimes my thoughts are like...I probably keep them to myself a little bit. You know, stressful things, I probably wouldn’t share them with my partner so much.” (C12, Patient)  “Normally, when I go through stressful moments, I can be very kind of irrational, you know, very instant. I wouldn't sort of wait and think about it. I’d just be very ‘Ahhhh!’ You know, very reactive, very stressed.” (C12, Partner) |
| Acceptance - accept what already happened | “Not really. You know, I just face things when they come. There’s nothing I can do to prevent it, so I’m not going to spend today worrying about something I can’t control.” (C17, Patient)  “Yeah, I remember it well. I’ve always been a very accepting person. My approach is that you deal with things the best way you can.” (C17, Partner) |
| Active coping - commonly used coping strategies | “My coping strategies? I’m not sure I have the best approach, but I tend to go quiet for a few days and withdraw from things. Eventually, something, a conversation, an event, or an interaction, will bring me out of it. I used to enjoy painting, and it was very relaxing.” (C11, Patient)  “Well, I’d like to say alcohol [laughing]. But we’re doing dry January, so. I don't know, occupy no minds, going for a walk, talking. Yeah, we get out of the house.” (C11, Partner) |
| Change in intimacy - changes in physical intimacy | “Well, the physical side of it, we’ve not been able to do anything for quite a number of years because of all that was running up to that.” (C1, Patient)  “Yeah, since he had his issues with his prostate, and he had the urethra tubed. He hasn’t been able to get an erection, so the sexual side of our relationship is no longer there.” (C1, Partner) |
| Relationship re-evaluation - how couples perceive the importance of their relationships | “Yes, I think knowing how much I care about my relationship can help me to cope because if you’re open with each other, you’ve no secrets. When you have a situation that happens to you, I think it helps you to cope better.” (C3, Patient)  “Yes, very much so. Our relationship means a lot to me. I think it’s much harder to cope if you don’t have a deep relationship or if you’re not open to talking about it.” (C3, Partner) |
| Reconnection - how cancer experience brings couples together and closer | “So, I think I’m very close now, probably a little bit closer in some ways to my wife, but I believe that as a partner, you know, we can work at this.” (C7, Patient)  “It is different, but in some ways, I would say we’re actually closer. I mean we’ve always been a really good couple. But I think not just with his cancer, but on you know on top of sort of other life events as well. You know I think we’ve grown really strong between us and it has in some ways it’s really enhanced our relationship, you know, because we have really deep conversations now.” (C7, Partner) |
| Physical challenges - challenges related to physical abilities | “Alongside that, the radiation side effects, particularly urinary problems, haven’t affected things much at home or in our relationship directly. But they do impact plans when we’re going out or going on holiday. I have to think ahead about what I need to take with me, and that part still feels like a negative effect.” (C5, Patient)  “I don’t know what my husband has said, but it has affected his incontinence. I think my husband feels upset about that. Yeah, it’s not easy for him, obviously.” (C5, Partner) |
| Emotional challenges - challenges related to emotional difficulties | “Well, mental challenges. I mean, obviously part of being male is being able to get an erection. It doesn’t bother [Partner 2], as far as I know. It bothers me more because I think that perhaps she’s missing out on something.” (C2, Patient)  “I suppose I know [Patient 2] gets upset sometime. Upset that’s the one word he gets annoyed, if he doesn’t get climax quickly or whatever.” (C2, Partner) |
| Social challenges - challenges related to social life | “We do share our thoughts, but sometimes, rightly or wrongly, my wife and I don’t always agree. We’re two different people, after all. When that happens, one of us might go off to another part of the house for a bit. But that’s just part and parcel of a relationship.” (C6, Patient)  “When we have a difference of opinion. Yeah, we have some good differences of opinion sometimes, yes, yes. I sulk for a bit, and then he’ll come and say, ‘Isn’t it about time you stop sulking?’ You know, wouldn’t all be a horrible place if we all thought the same and did the same?” (C6, Partner) |
| Partner identity - how couples describe themselves | “Probably not the best. Like many men, I think I’m a man. I say things, and she takes offence at them. I think, what have I said? No, I’m definitely not the perfect partner.” (C10, Patient)  “Probably annoying, haha. On the whole, I think I’m quite caring. I do look after the house. You know, I do all the washing, the tidying, and all that kind of stuff. So, I think we’ve got a stable routine. And I think I probably put in that routine quite a lot.” (C10, Partner) |
| Partner values - what couples value the most their relationships | “I think the trust and lovingness between us. I can tell her everything and anything.” (C18, Patient)  “It really comes down to honesty. I can trust him, and he can trust me. We tell each other everything, and that’s really important.” (C18, Partner) |
| Relationship goals - what goals couples want to achieve | “Just to continue being there for each other and understanding each other.” (C9, Patient)  “Well, I suppose it’s about trying to be more open with each other. Maybe we haven’t always been as open as we could be, and it would be nice to be able to share more honestly.” (C9, Partner) |
| Daily activities - what things couples do on a daily basis | “We cook together when needed, and we go shopping together. We also look after our grandchildren twice a week, always as a team. If we're visiting someone, we do it together.” (C16, Patient)  “Well, in terms of daily activities, we often cook together and look after the house together. We’re very much involved with veterans’ groups, so we attend those meetings and visit friends and family. We also do the shopping together.” (C16, Partner) |
| Planning - making plans about things couples want to do | “Oh, yeah. We’re off on a holiday together, the whole family, very soon. We’re always planning the next holiday or family gathering, so there’s always something to look forward to.” (C14, Patient)  “Yes, holidays are a big thing for us. We love to travel, and we’re about to go on a five-week holiday.” (C14, Partner) |
| Partner support - things partners do to support each other | “I worked as a chef in my career, so I try to share the burden. Right now, she’s got a really nasty cold, so I’ll go and do the shopping, cook the meals, and do whatever’s needed to help keep the household running.” (C16, Patient)  “I’m always there, listening and asking questions. Not just about the cancer, but about life in general.” (C16, Partner) |
